# Bide Your Time With Bites: A Case of Rattlesnake Envenomation in Pennsylvania

**DOI:** 10.7759/cureus.66539

**Published:** 2024-08-09

**Authors:** Xavier Zonna, Conor W Banta, Ronald Lott, Shweta Shah, Anthony Battista, Caroline Colleran

**Affiliations:** 1 Internal Medicine, University at Buffalo, Buffalo, USA; 2 Internal Medicine, Geisinger Commonwealth School of Medicine, Scranton, USA; 3 Internal Medicine, Lake Erie College of Osteopathic Medicine, Erie, USA; 4 Family Medicine, UPMC Shadyside, Pittsburgh, USA; 5 Emergency Medicine, Saint Vincent Hospital, Erie, USA

**Keywords:** swelling, antivenom, coagulopathy, evenomation, rattlesnake

## Abstract

This case report represents a 26-year-old male with no significant past medical history who presented to the emergency department in western Pennsylvania following a western diamondback rattlesnake (*Crotalus Atrox*) bite to his hand. His initial swelling was mild, and his coagulation studies were unremarkable, with minimal changes on repeat studies, and poison control recommended against antivenom administration. He was discharged home with oral antibiotics and analgesics due to his stable clinical course. However, he returned to the emergency department about 12 hours later with worsening pain and swelling that extended to his elbow. He was then given antivenom and transferred to a larger center for higher-level care, ultimately having symptom resolution after further antivenom administration. This report serves to underline the importance of clinician education regarding envenomation management throughout the United States, including areas without indigenous venomous snakes.

## Introduction

Medical treatment of venomous snake bites presents a challenge to emergency room physicians worldwide because of their complexity in management and variability in clinical course. Each year in the United States, there are approximately 10,000 emergency department (ED) visits for snake bites, of which about a third are estimated to result from venomous snakebites [1]. Venomous snakes are further subdivided into pit vipers and coral snakes [2]. Pit vipers including rattlesnakes are far more common in the Southwestern and Western regions of the United States [2]. This habitat distribution leads to a paucity of cases in the Northeast of the United States. Although rare, venomous snakebites can lead to a variety of cytotoxic, neurotoxic, and hemotoxic complications that may progress to become life-threatening [2].

In 2013, The American College of Medical Toxicology established the North American Snakebite Registry (NASBR), a database that compiles, categorizes, and conveys information related to snake envenomations in the United States. Currently, 19 major institutions in the US report snake envenomations to the NASBR. Historically, the vast majority of snake envenomations occur in the Southwestern US and are from native pit viper snakes (rattlesnakes, copperheads, cottonmouth), usually affecting men (69.3%) with bites in the lower extremity (54.2%) [3]. For rattlesnake bites in particular, swelling was reported in 94.5% of cases, ecchymosis in 59.4%, erythema in 42.6%, and necrosis in 7.8% [3]. Furthermore, NASBR data from 2013 to 2015 demonstrates the highest incidence of initial serious hematologic complications such as thrombocytopenia (16.4%), hypofibrinogenemia (17.2%), and coagulopathy (32.8%) in patients envenomated by rattlesnakes when compared to the incidence in copperhead and cottonmouth snakes [3].

This report details a case of western diamondback rattlesnake (*Crotalus atrox*) envenomation to the upper extremity requiring anti-venom treatment in a large Northwestern Pennsylvania city. His clinical course was prolonged because of a premature hospital discharge, leading him to present back to the emergency department several hours later. Despite the serious sequelae of snake envenomation, most providers are not experienced in treating these patients [3]. Furthermore, the data regarding ideal treatments for these patients is severely limited [3]. The goal of this report is to systematically analyze the assessment and treatment process of venomous snake bites to find a standardized management strategy. It is also to illustrate the importance of understanding snake envenomation management and complications even in areas without endemic venomous snakes and to raise awareness about the serious and potentially morbid consequences for such patients.

## Case presentation

**Figure 1:**
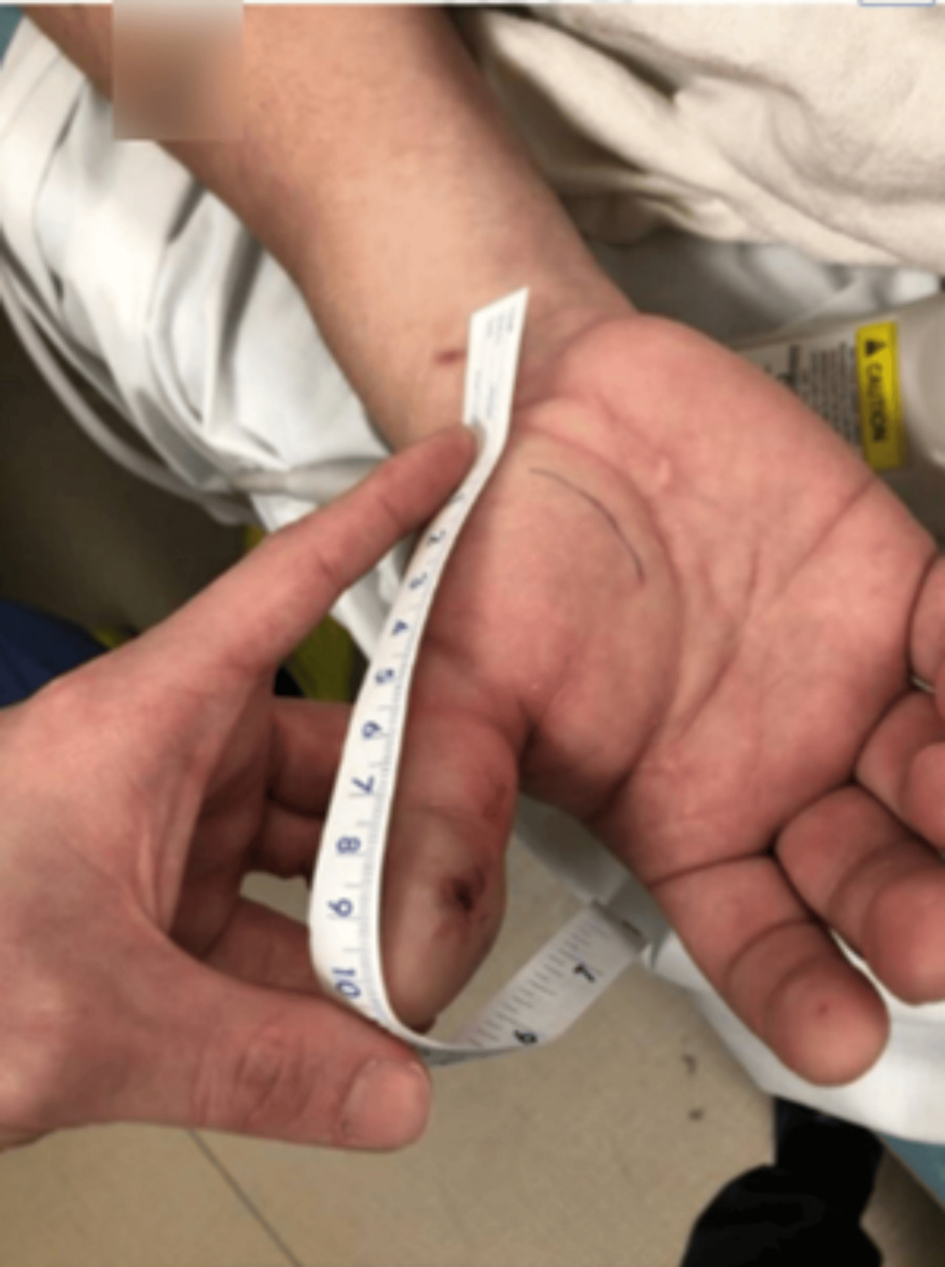
Right volar hand on initial presentation

A 26-year-old male with no significant past medical history presented to the ED at 6:52 p.m. complaining of hand pain after sustaining a puncture wound from a western diamondback rattlesnake (*Crotalus atrox*) one hour prior while attempting to clean the snake’s enclosure. He stated that he had digital paresthesia en route to the emergency room but reported no paresthesia on admission. On physical examination, there was mild swelling and erythema of the right first digit with intact strength and sensation of the hand. At this time, swelling and erythema were localized to the right first digit and thenar eminence. Poison control was contacted and recommended coagulation studies to evaluate the need for antivenom administration. This patient’s initial internal normalized ratio (INR) at 7:24 p.m. was 1.0 (normal: 0.8-1.1), prothrombin time (PT) was 13.2 seconds (normal: 11-13.5 seconds), partial thromboplastin time (PTT) was 33 seconds (normal: 25-35 seconds) and fibrinogen was 313 mg/dL (normal: 200-400 mg/dL). At 11:17 p.m., INR increased to 1.1, PT increased to 14.4 seconds, PTT increased to 38 seconds, and fibrinogen remained stable at 314 mg/dL. ED physicians spoke with poison control who recommended no antivenom at the time due to the length of time elapsed (five hours) since the bite with such a small change in coagulation studies. The patient was discharged seven hours after the initial bite with an amoxicillin/clavulanate prescription for the bite wound and acetaminophen/hydrocodone for pain with advice to return to the ER should any cyanosis, necrosis, numbness, or bleeding develop. At the time of discharge, swelling was limited to the thenar eminence and distal phalanx (Figure 1).

**Figure 2:**
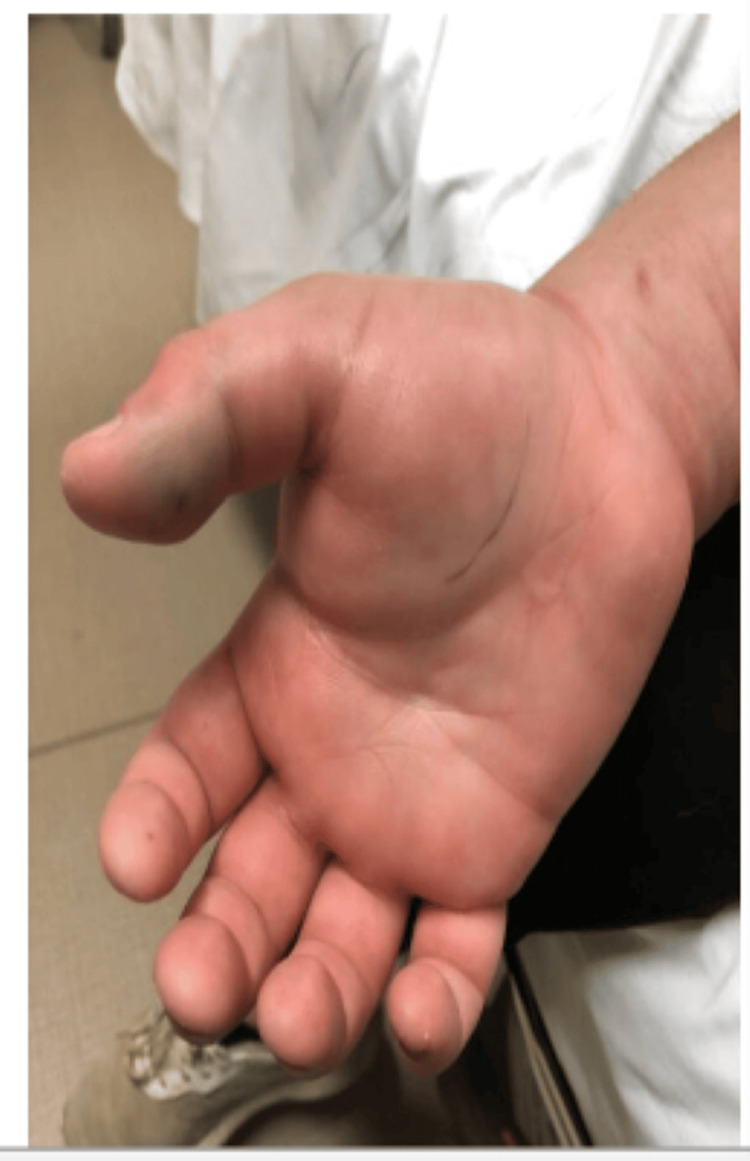
Right volar hand the following morning

**Figure 3:**
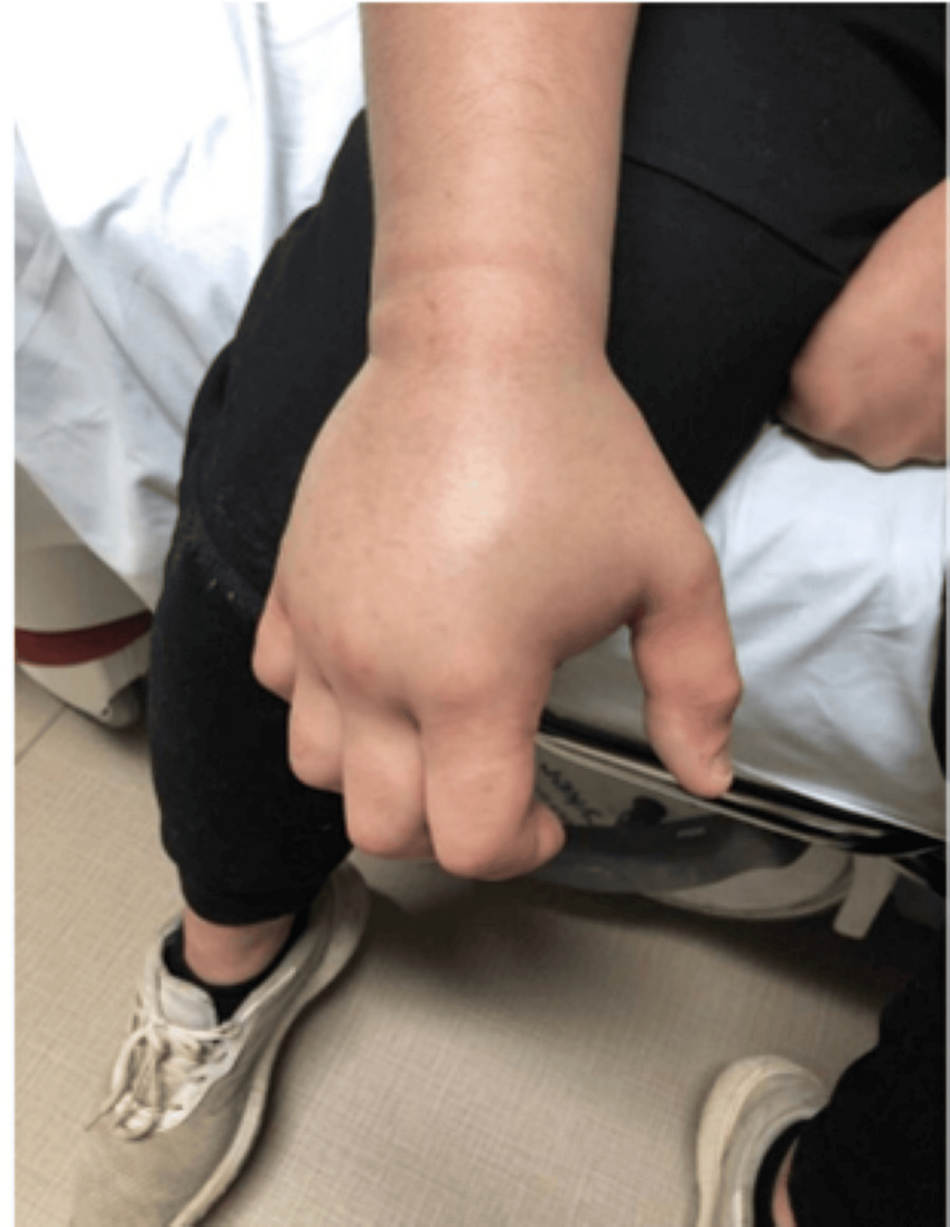
Right dorsal hand 15 hours after initial presentation

**Figure 4:**
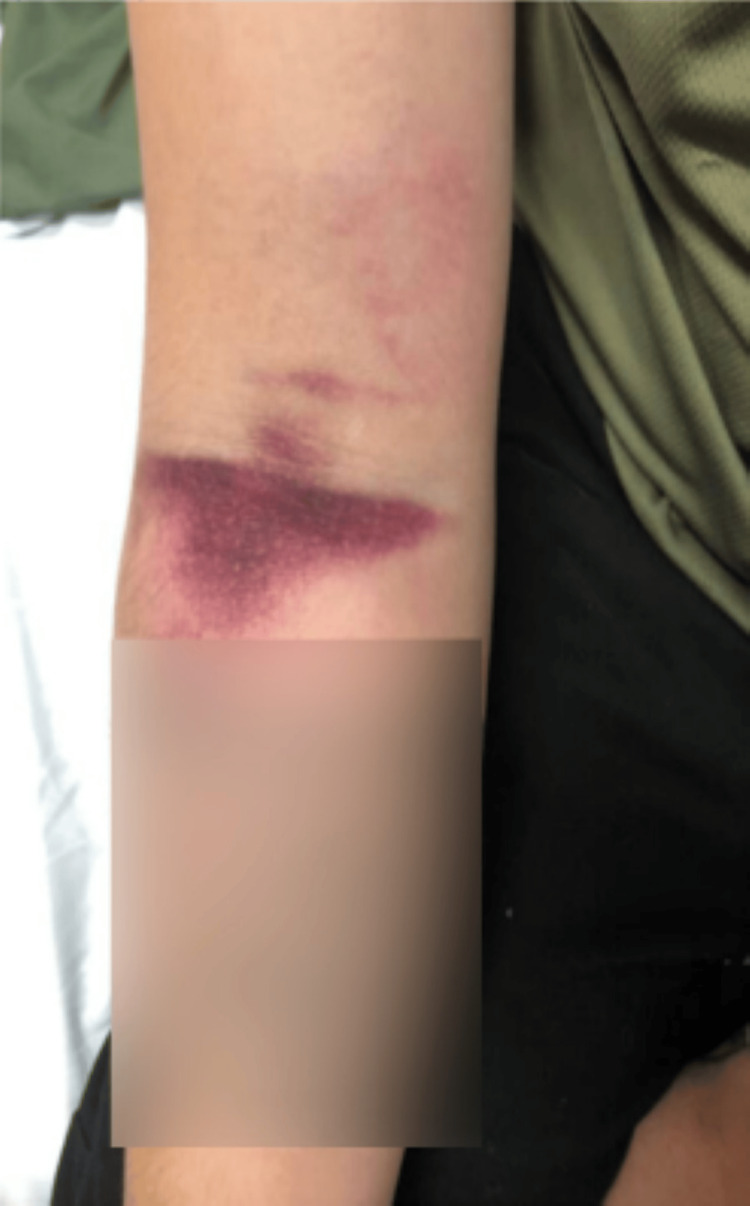
Right ventral forearm 15 hours after initial presentation

The patient then returned to the ER later that morning 15 hours after the initial presentation with worsening swelling and pain. He stated that the acetaminophen/hydrocodone and ibuprofen were not sufficiently controlling his pain. A review of systems was negative for any new symptoms including chest pain, shortness of breath, vomiting, nausea, or paresthesia. On physical exam, the swelling had extended, now encompassing the entire right hand and forearm stopping at the elbow with ecchymosis of the right cubital fossa (Figures 2-4).

Toxicology at a larger center was consulted, and they recommended repeating blood work given the increase in swelling, starting the patient on six vials of Crotalidae polyvalent immune Fab antivenin, and keeping the arm raised while a transfer was arranged for further evaluation. Repeat coagulation studies revealed D-Dimer 2.41 mg/L (normal: 0-0.50 mg/L), INR 1.1, PT 14.0 seconds, PTT 38 seconds, and fibrinogen 325 mg/dL. Upon transfer, the patient was placed on a maintenance dose of antivenom, tolerating it well with improving swelling and pain levels with serial circumference checks of the extremity. He was determined to have no blood dyscrasias with serial lab work and was deemed stable for discharge with close follow-up. Toxicology recommended against using non-steroidal anti-inflammatory drugs (NSAIDs) for pain control due to their potential to increase bleeding risk.

## Discussion

ED management of venomous snake bites in the United States is relatively rare, with recent Center for Disease Control (CDC) estimates of about 7,000 venomous snake bites occurring annually [4]. Although these bites occur throughout the United States, they are disproportionately common in the Southwest where such venomous snakes are indigenous [2]. Pit vipers are responsible for the majority of the envenomations in the United States [3]. The management of such snake bites is widely variable, largely due to the inherent heterogeneity of snake bites and the differential amount of venom instilled in victims as well as natural variations in the chemical composition of such venoms [5]. Some studies have suggested that venom toxicity and composition can vary significantly even between snakes of the same species simply based on geographic location of snake capture, with western diamondback rattlesnakes from the Southeast United States showing the highest lethality in mice of any region of capture and those from the Northeast showing the lowest [5]. This difference in venom efficacy is unlikely to be related to diet and environment, meaning that snakes in captivity should not be expected to have significantly different venom composition or effects as compared to wild snakes [6].

A study of the combinatorial peptide ligand libraries with electrophoresis and mass spectrometry of the *Crotalus atrox* venom identified a variety of enzymes, primarily metalloproteinases and serine proteases, thought to be responsible for the cytotoxicity, hemotoxicity, and myotoxicity of such venom [7]. Gutierrez and Rucavado explored the metalloproteinases in this venom, explaining that they likely cause damage directly to the basement membrane and extracellular matrix, leading to hemorrhage with fluid extravasation and localized inflammation; subsequent bleeding and reductions in muscle perfusion then cause further myonecrosis with fibrosis impairing future capacity for cellular regeneration [8]. Phospholipase A2 enzymes also contribute to the toxicity of snake venom across a variety of snake species including the Crotalidae family [9]. Xiao et al. explain that phospholipase A2 causes neurotoxicity by presynaptically blocking neuromuscular transmission in addition to causing myotoxicity through the disruption of cell membranes and calcium channels as well as coagulopathic effects through direct factor X inhibition and disrupted platelet aggregation resulting from the hydrolyzation of necessary phospholipids [9].

Despite the perceived risk for infection in bite/puncture wounds, a low percentage of patients in research receive antibiotics for infection prophylaxis after snake envenomations [3]. The bacterial composition of snakebite envenomations was studied by Senthilkumaran et al., finding that these envenomations were usually monomicrobial, with the most frequent bacteria being *Staphylococcus aureus*, *Klebsiella *sp, *Pseudomonas aeruginosa*, and *Escherichia coli *[10]. Despite identifying common bacteria that are treatable with easily accessible antibiotics, authors have repeatedly recommended against the use of antibiotic prophylaxis for snakebite envenomations in otherwise healthy adults due to the low (<5%) incidence of post-bite infections [11-12]. A literature review of articles regarding antibiotic prophylaxis in snakebites came to a similar conclusion, recommending against antibiotic prophylaxis for all except immunocompromised patients or those with complicated wounds [13]. In a NASBR study involving 450 cases over three years, only two patients had confirmed infections during hospitalization indicating that antibiotic administration is largely unnecessary [3].

Management of venomous snake bites is multifaceted, with proper treatment involving monitoring and addressing issues across a variety of bodily systems, from coagulation and hemodynamics to local tissue destruction and necrosis. In 2011, Lavonas et al. created a unified treatment algorithm for the management of crotalid snake bites in the United States [14]. In this algorithm, they emphasized the importance of initial patient evaluation with baseline laboratory studies, including coagulation studies, fibrinogen, and a complete blood count followed by monitoring patients’ clinical and laboratory progression for at least 12-24 hours before discharge for even mild presentations [14]. These treatment guidelines also challenged what many physicians would consider to be best practices for analgesia, recommending opioids over NSAIDs due to the concern for platelet dysfunction increasing bleeding risk, although this does not have extensive supportive evidence [14]. In addition to this general management, consideration of the anatomical location of snakebites is important for their management, particularly in the context of progressing inflammation. For instance, Hwang and Flach described a patient with a right upper extremity snakebite that required emergent cricothyrotomy to maintain airway patency following oropharyngeal edema in addition to requiring an intravenous antivenom drip for persistent coagulopathy [15].

The coagulopathy resulting from snakebite envenomations should not be underestimated, especially considering the extended time frame over which it may present, with one case report finding clinically relevant coagulopathy more than two weeks after initial envenomation [16]. Dudley et al. explained that coagulopathies following snake bites are probably significantly underdiagnosed and underreported [17]. In their 2022 study, they compared late coagulopathies in patients after being treated with two of the most common antivenoms in the United States, Crotalidae polyvalent immune Fab and Crotalidae immune F(ab')2, finding that Crotalidae immune F(ab')2 may be associated with decreased late onset coagulopathy in some patients [17]. Few studies have examined the long-term effects of snake bites on quality of life and symptom burden, although one population-based study found significant long-term complications in over 13% of snake bite victims who reported an average symptom duration of 12 years from initial envenomation [18].

Due to the rarity of ED visits for snake bites, the decision to use antivenom is multifactorial and can be controversial, differing considerably throughout the available literature. A current limitation of snake bite standardization is the lack of large-scale trials with the administration of antivenom due to limited data [3]. A study examining pediatric snake bites in South Texas concluded that significant morbidity was uncommon and that indications for the usage of antivenom necessitate further research [19]. A retrospective cohort study examined the use of antivenom for Copperhead snake bites and concluded that factors determining antivenom administration were younger age, symptoms crossing major joints, and upper extremity bites [20]. These authors also recognized the need for a systematic methodology to determine the need for antivenom in patients [20]. Lavonas et al. recognized this need and attempted to standardize the management by performing a literature review and database analysis [14]. They determined that indications for the use of antivenom include progressive local effects, coagulopathy, or systemic signs and symptoms of toxicity while recommending against antivenom administration in patients with only localized swelling and pain at the site of the bite [14]. These authors also discussed that swelling crossing major joints or significant progression can be an indication for antivenom administration in addition to significantly deranged coagulation studies during observation [14]. In the case described above, this patient’s affected site was initially limited to the hand (Figure 1) but then extended to the wrist and progressed toward the elbow necessitating antivenom administration (Figures 2-4). With initial local signs of envenomation, observation of 12-24 hours is recommended with administration of antivenom for progression of effects [14]. This patient’s in-hospital observation time of approximately six hours was inappropriately short and extended observation was warranted which would have allowed for more timely treatment administration once his extremity swelling expanded and increased in severity.

## Conclusions

Due to their rarity, variability in presentation, and narrow geographic distribution, venomous snake bites are not commonly encountered by many emergency department healthcare providers throughout the United States. These challenging characteristics are further compounded by the breadth of available treatment options, ranging from simple observation to rapid antivenom administration and invasive surgical intervention. Yet, standardized treatment algorithms and plans of care are not widely implemented for these patients. As a result, patients seeking treatment for snake envenomations face a highly variable quality of care. As evidenced by this case, bites from the Western diamondback rattlesnake necessitate close observation and timely antivenom administration for significant envenomations to avoid premature patient discharges, systemic toxicity, morbidity, and mortality. This case highlights the current paucity of organized decision-making for snake envenomations at multiple levels of care, from local emergency departments to regional poison control centers, resulting in a striking care gap for some snake bite patients. Moving forward, improving patient experiences and outcomes by addressing this care gap will require greater uniformity in provider education and the broad implementation of fundamental treatment algorithms for the initial triage and management of these patients.
